# Rating of physiotherapy student clinical performance in a paediatric setting: are assessors consistent in their rating of a simulated clinical student performance?

**DOI:** 10.1186/s12909-023-04149-9

**Published:** 2023-04-24

**Authors:** Tessa Fulton, Kerry Myatt, Garry W Kirwan, Courtney R Clark, Megan Dalton

**Affiliations:** 1 Physiotherapy Department, Queensland Children’s Hospital, South Brisbane, QLD 4001 Australia; 2 Physiotherapy Department, QEII Jubilee Hospital, Metro South Health, Coopers Plains, QLD 4109 Australia; 3 Menzies Health Institute, School of Health Sciences and Social Work, Griffith University, Gold Coast Campus, 4222 Australia; 4 Griffith Institute for Educational Research, Griffith University, 1 Parklands Drive, Gold Coast Campus, Southport, QLD 4222, 4215 Australia; 5 School of Physiotherapy, Australian Catholic University, Brisbane, Australia

**Keywords:** Clinical education, Physiotherapy, Paediatrics, Assessment, APP

## Abstract

**Background:**

During workplace-based clinical placements, best practice assessment states students should expect consistency between assessors rating their performance. To assist clinical educators (CEs) to provide consistent assessment of physiotherapy student performance, nine paediatric vignettes depicting various standards of simulated student performance, as assessed by the Assessment of Physiotherapy Practice (APP), were developed. The APP defines adequate on the global rating scale (GRS) as the minimally acceptable standard for an entry-level physiotherapist. The project aimed to evaluate consistency of paediatric physiotherapy educators assessing simulated student performance using the APP GRS.

**Methods:**

Three paediatric scenarios representing neurodevelopment across three age ranges, infant, toddler and adolescent, were developed and scripted that depicted a ‘not adequate’, ‘adequate’ and ‘good-excellent’ performance based on the APP GRS. An expert panel (n = 9) conducted face and content validation. Once agreement was reached for all scripts, each video was filmed. A purposive sample of physiotherapists providing paediatric clinical education in Australia were invited to participate in the study. Thirty-five CEs, with minimum 3-years clinical experience and had supervised a student within the past year, were sent three videos at four-week intervals. Videos depicted the same clinical scenario, however performance varied with each video. Participants rated the performance on the four categories: ‘not adequate’, ‘adequate’, ‘good’ and ‘excellent’ Consistency among raters was assessed using percentage agreement to establish reliability.

**Results:**

The vignettes were assessed a combined total of 59 times. Across scenarios, percentage agreement at the not adequate level was 100%. In contrast, the adequate scenarios for the Infant, Toddler and Adolescent video failed to meet the 75% agreement level. However, when combining adequate or good-excellent, percentage agreement was > 86%. The study demonstrated strong consensus when comparing not adequate to adequate or better performance. Importantly, no performance scripted as not adequate was passed by any assessor.

**Conclusions:**

Experienced educators demonstrate consistency in identifying not adequate from adequate or good-excellent performance when assessing a simulated student performance using the APP. Recommendation for practice: These validated video vignettes will be a valuable training tool to improve educator consistency when assessing student performance in paediatric physiotherapy.

**Supplementary Information:**

The online version contains supplementary material available at 10.1186/s12909-023-04149-9.

## Background

Quality clinical education in paediatric physiotherapy is integral to the development of competent health professional graduates. In physiotherapy programs across Australia and New Zealand, students are assessed on their ability to deliver entry-level physiotherapy services to the paediatric population across a variety of clinical settings. The minimally competent standard is defined within the Physiotherapy Practice Threshold Statements [1]. However, to assess performance effectively, consistency independent of the clinical area and setting is essential to maintain grade integrity [2, 3].

Grade integrity defines a grade as “representing the quality, breadth and depth of the level of achievement a student reaches” [4]. Essentially the grade applied to a student performance is representative of the actual performance and so that grade is able to accurately determine the level of competence of the student. Competence is an ongoing state and therefore assessment needs to include actual performance as well as the demonstrated ability to adapt to change and seek new information [5]. Within the paediatric setting, physiotherapy students are assessed using the Assessment of Physiotherapy Practice (APP) [6][7]. The APP uses 20 items divided into seven domains of practice (professional behavior, communication, assessment, analysis and planning, intervention, evidence based practice and risk management) that are assessed on a 5-point scale (0–4), where a score of two is defined as the minimally competent standard to enter the profession [8]. The APP has a separate global rating scale (GRS), that uses the following 4 categories of ‘not adequate’, ‘adequate’, ‘good’ and ‘excellent’. The APP defines an adequate on the global rating scale (GRS) as the minimal acceptable standard for an entry level physiotherapist. A non-adequate rating indicates the student did not demonstrate the minimal acceptable standard and a good or excellent rating will indicate that the student performance was at a level above the minimum standard. However, anecdotally clinicians within the paediatric setting report additional challenges to the interpretation of the APP due to the nuances of the paediatric environment especially in regard to what may be considered an adequate student performance. Furthermore, there is a lack of resources and training to support this clinical setting.

Evidence supports the benefit of consensus moderation to deliver consistent, accurate and effective assessment [9]. This is supported by the fact that learning resources related to assessment using the APP are available for other clinical settings. A study conducted by Kirwan et al. (2019) demonstrated the potential variability that may exist in clinical assessment, mostly in determining an adequate performance, and how training and resources using video vignettes can augment assessment practices.

In response to a gap in the literature, the aim of this study was to determine the level of consistency among paediatric clinical educators when assessing a simulated student performance using the APP GRS.

## Methods

Ethical approval was granted by The Human Research Ethics Committee of Queensland Children’s Hospital (protocol number - HREC/16/QRCH/362) and Griffith University (protocol number 2016/941) prior to the study’s commencement. All methods were carried out in accordance with the relevant guidelines and regulations. Informed consent was obtained from all participants as per the ethics guidelines.

### Development of the vignettes

Three paediatric scenarios representing the core area of neurodevelopment across three age ranges, infant, toddler and adolescent, were developed and scripted into performances that depicted a ‘not adequate’, ‘adequate’ and ‘good to excellent’ performance based on the APP GRS. The GRS was used in lieu of scoring in each domain on the whole APP as not all domains may be shown in an individual performance and so this may skew the results of the performance results if participants rated what they didn’t see as a 0 on the APP. The scenarios included an infant with failure to thrive, a pre-school aged child with developmental coordination disorder and an adolescent with Cerebral Palsy. An expert panel of clinical educators (n = 9), experienced in one of the three areas of paediatrics mentioned, sought from paediatric hospitals and universities within Australia, was convened to conduct face and content validation. Each panel member independently reviewed each of the three performance levels from their clinical area of expertise (n = 3 scripts per panel member). Panel feedback on the level of performance, clinical authenticity and accuracy of the script was collected and recommendations to improve the face and content validity were collected for incorporation into the final scripts.

Once all experts reached agreement for all nine scripts, each video was filmed using a standardised actor to portray the student physiotherapist. Children who portrayed patients during filming were known to the project team and consented to participate. This included one child with a known neurological condition and two children who were typically developing. Clinical staff from Queensland Children’s Hospital played the role of clinical educator and parent. During the filming of each scenario, the authors were present to direct each scene to ensure adherence to the script. Each video was on average 18 min in length.

### Assessment of reliability

A purposive sample of physiotherapists providing paediatric clinical education in Australia were invited to participate in the study. The inclusion criteria were a minimum three years clinical experience in paediatrics and 1-year experience in the clinical education of physiotherapy students. Participants who did not meet the inclusion criteria were excluded to ensure the sample population was familiar with the assessment of student performance using the APP.

Paediatric physiotherapists identified as meeting the inclusion criteria were invited to participate via email correspondence. Participants were provided with a Participant Information sheet detailing their involvement including the nature of the study and the total time commitment. Consent to participate was achieved via an ‘opt in’ approach with a response to the sent email indicating consent to participate. Consent was confirmed from the participants prior to completing the study survey (Supplementary 1). Participants included in the study were allocated to a clinical scenario in their nominated area of expertise (infant, toddler or adolescent).

Following provision of consent and group allocation, participants were emailed detailed directions for reviewing the video vignette and completing the evaluation via Survey Monkey®. Each participant was sent a total of three videos vignettes, representing ‘not adequate’, ‘adequate’ and ‘good to excellent’, over a 12-week period of time. The vignettes were sent in a randomly allocated order to minimise bias. At the completion of the first vignette, participants were asked to provide demographic information in addition to a global rating scale based on the APP, key factors used to determine the global rating and feedback on video quality and clinical relevance that was collected for all three video vignettes. Vignettes were securely stored on Google Drive and distributed by email in the form of a closed link.

Participants were required to independently watch and complete a survey for three video vignettes in the same clinical area. A wash out period of four weeks between the sending of each video was selected to ensure that it was unlikely that participants would be able to recall specific information of the previously watched video [10, 11].

The video and survey links were closed two weeks after the initial email. After a 4 week wash out period, the same process was repeated with the second video with a new video and survey link. To maximize response, a reminder email was sent to all participants one week after the initial email. Furthermore, all participants who completed the study by watching all three videos were provided a financial incentive equating to $50AUD.

### Data analysis

Data from SurveyMonkey® was extracted as a comma-separated value (CSV) file and converted to Microsoft Excel^™^ (Microsoft Corporation, Redmond WA). The data was cleaned and separated into percentage agreement, demographic, key performance indicators (KPIs) and video vignette evaluation data. Percentage agreement was calculated for each of the nine videos using SPSS 21.0 software package**®** (SPSS Inc., Chicago, IL, USA) and a priori agreement of 75% was considered acceptable as per the previous study [8].

## Results

### Participant Demographics

Forty-three clinicians across various clinical settings were identified as meeting the inclusion criteria and invited to participate, of which 39 consented to participate. Four participants (11%) withdrew from the study citing time constraints (n = 3) and technical issues (n = 1) as primary reason. Seventeen (48.6%) completed more than one video and 7 went on to complete all three videos assessments (20%). In total, the suite of videos was viewed a total of 59 times for the purposes of data collection.

**Fig. 1:**
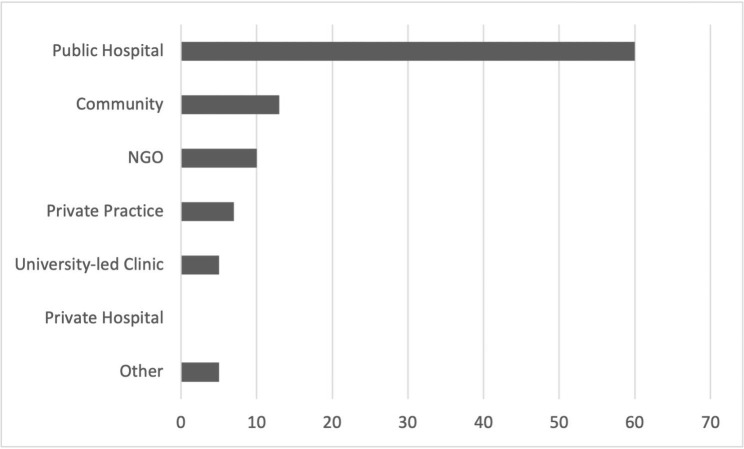
Title: Primary clinical setting of participants; X: Percentage; Y: Primary clinical setting; Legend: Distribution of Primary clinical setting of respondents

Responses were received from participants in four states of Australia (Queensland = 60%, Victoria = 26%, NSW = 9%, and Western Australia = 6%). Most participants reported working in the public hospital setting (66%), however there was representation from a variety of clinical settings as outlined in Fig. 1. 69% of participants worked in a metropolitan area, with 29% regional and 3% rural/remote. Participants reported a range of clinical experience in the field of paediatric physiotherapy, as well as their paediatric clinical education experience (Table 1). 97% of participants reported a confidence level of ‘somewhat confident’ or greater with 80% being confident or very confident in using the APP to assess student performance.

**Table 1:** Participant characteristics

Participant Demographics	Frequency (n)	Percent (%)
Clinical Experience (n = 35)		
3–5 years	2	5.7
6–8 years	7	20
9–11 years	6	17.1
12–15 years	4	11.4
More than 15 years	16	45.7
Experience as an Educator (n = 35)		
1–3 years	7	20
4–6 years	11	31.4
7–9 years	5	14.3
10–12 years	3	8.6
12–14 years	2	5.7
More than 14 years	7	20
Confidence in using the APP (n = 35)		
Not confident	1	2.9
Somewhat confident	6	17.1
Confident	24	68.6
Very confident	4	11.4

*APP – Assessment of Physiotherapy Practice*

### Percentage Agreement

A total of 59 complete responses were collected across the nine video vignettes and included for analysis.

Percentage agreement for the Infant, Toddler and Adolescent videos at the not adequate and good-excellent level achieved greater than the 75% agreement. In contrast, the adequate scenarios for the Infant, Toddler and Adolescent video failed to meet the 75% agreement level (Table 2). However, when analysing the adequate videos by comparing not adequate to a combined rating of adequate and good-excellent, percentage agreement increased to > 75% in all categories (Table 3). Only one assessor rated the adequate scenario for the Adolescent as not adequate, no assessor rated the infant or toddler adequate video as not adequate.

**Table 2:** Scenarios and percentage agreement with APP GRS

		BLIND ASESSMENT				
		Not Adequate	Adequate	Good	Excellent	Agreement
INFANT	Not Adequate	5	0	0	0	100%
	Adequate	0	4	1	2	57.10%
	Good-Excellent	0	0	3	6	100%
TODDLER	Not Adequate	4	0	0	0	100%
	Adequate	0	6	2	1	66.66%
	Good-Excellent	0	0	5	2	100%
ADOLESCENT	Not Adequate	6	0	0	0	100%
	Adequate	1	0	4	2	0%
	Good-Excellent	0	1	0	4	80%

**Table 3:** Adequate scenario percentage agreement with APP GRS

		Not Adequate	Adequate and Good-excellent	Agreement
Adequate	Infant	0	7	100%
	Toddler	0	9	100%
	Adolescent	1	6	86%

### Evaluation of video vignettes

All participants either agreed (56%) or strongly agreed (44%) that the clinical scenario they viewed were realistic and believable. Most participants (98%) reported that the clinical scenario was professional and well presented.

## Discussion

The aim of the project was to undertake a reliability study for a suite of video vignettes depicting paediatric physiotherapy student performance based on the APP GRS. The study demonstrated acceptable consensus among participants at the not adequate and good to excellent level. However, at the adequate level there was insufficient exact agreement. If ratings of adequate and good-excellent were combined consensus once again reached an acceptable level when comparing ‘not adequate’ to ‘adequate’ or better.

In line with previous research investigating consensus in the adult clinical setting, the study showed strong agreement in rating the not adequate and the good-excellent performance across all three clinical scenarios [8]. This is relevant for clinical practice, particularly in relation to the level of agreement when rating the not adequate videos. While the GRS does not count towards the overall score on the APP, it is used to assess the alignment with the overall marking, providing a holistic view of the student performance, rather than looking at each domain on its own. Therefore, understanding what an overall adequate performance is, is vital in understanding minimum competency. Furthermore, consistency in scoring students correctly using the APP is essential for ensuring all pre-registration students meet an adequate level of performance prior to entering the physiotherapy profession [7]. In this study, all not adequate performances were correctly identified by participants, and only one adequate performance received a not adequate rating.

There was insufficient consensus for the adequate video scenarios, which is a similar outcome to the previous adult video vignettes research [8]. Simulated student performance at both the not adequate and excellent levels is more easily recognised and rated by assessors whereas the variability in students performing at an adequate standard makes assessment at this level more difficult. Assessors may have found the rating of “adequate’ more difficult due to the relatively short length of the video performance (average length across all three scenarios was 18.35 minutes), and that educators were not able to observe students’ repeat performances across multiple patients as they would do in traditional longitudinal clinical placements, participants may have missed important aspects of the student’s performance and thus influenced their rating using the APP GRS. As per previous results, an ‘adequate’ performance may have its own variability within the performance, i.e., students may perform well in certain areas but underperform in others. If clinical educators themselves believe that particular areas have more weight than this may introduce inherent bias within the clinical educator who may be drawn towards certain performance criteria which if they are performed at a higher or lower level may lean the educator towards that level. Trede and Smith (2014) and Kirwan et al. (2019) reported similar findings within their studies showing clinical educator bias when conducting assessment or scoring on the GRS.

When adjusting the data in the adequate scenarios to compare an adequate or good-excellent versus a not adequate performance, strong consensus was achieved. This indicates that it is more difficult to differentiate an adequate performance from a good-excellent performance than from a not adequate performance. Our data shows that clinical educators with paediatric experience are able to differentiate competent and not competent student performance, when viewing a vignette.

However, as consistency has been achieved in determining a not adequate and good-excellent performance and the variability in scoring an adequate performance has been noted, these vignettes would be an important resource to educate clinical educators. In particular, the different levels of performance and the key criteria when assessing a student performance could be addressed.

## Limitations

While use of short video vignettes of simulated student performance is a recognised method used for consensus moderation and training of assessors to assess student performance during practical/OSCE examinations, determining if a student is performing at an adequate level in the clinical setting requires longitudinal assessment of performance [12, 13]. The simulated student performance was only rated using the APP GRS, and while a useful tool, students are not assessed using the GRS and on the sum total of all of the individual components of the APP. However, previous work done using vignettes showing student performance has shown this to be an effective tool to show global performance. Due to the nature of the paediatric physiotherapy population, only a small number of reviews were able to be completed as part of the data collection which limited the statistical analyses that could take place. Furthermore, only about half of the participants completed more than one video assessment.

## Conclusion

Experienced educators demonstrated consistency in identifying a not adequate from adequate or better performance when assessing a simulated student performance using the APP GRS. However, variability existed when assessing the ‘adequate’ performance with a lack of consensus in differentiating ‘adequate’ from ‘good/excellent’.

The resources developed for this body of research are freely available online (https://www.applinkup.com/Resources.aspx). A corresponding training package is available to be utilised by physiotherapists and universities for paediatric clinical educator support and training, student learning and development, and delivery of training and workshops.

## Recommendations for Practice

The developed suite of simulated student performance vignettes will be used as part of an already well established clinical educator training program. This training program assists clinical educators in determining appropriate levels of student performance in an adult population. This suite of vignettes can now be used for those working in a paediatric environment.

## Electronic Supplementary Material

Below is the link to the electronic supplementary material.

Supplementary 1: APP Vignettes Validity and Reliability Testing

## Data Availability

The datasets used and/or analysed during the current study are available from the corresponding author on reasonable request.

## List of abbreviations

CE: Clinical Educator
APP: Assessment of Physiotherapy Practice
GRS: Global Rating Scale
CSV: Comma Separated Value
KPI: Key Performance Indicator
