# Supplementary material for: Rating of physiotherapy student clinical performance in a paediatric setting: are assessors consistent in their rating of a simulated clinical student performance?
Source: BMC Med Educ. 2023 Apr 24;23:280. doi: 10.1186/s12909-023-04149-9 (PMC10127403; doi:10.1186/s12909-023-04149-9)
Supplement: Supplementary file 1 — Supplementary 1: APP Vignettes Validity and Reliability Testing [file 12909_2023_4149_MOESM1_ESM.pdf]

APP Vignettes Validity and Reliability Testing

Dear Participant

Thank you for taking the time to undertake a blind assessment of the APP vignettes to assist in building a resource to train and support clinical educators in the use of the APP.

All information is collected in a manner that is unidentifiable and completion of the survey will constitute consent. For complete information on the project please refer to the Participant Information Sheet attached to the email containing the link to this survey. If you did not receive a Participant Information Sheet then please contact Tessa Fulton on t.fulton@griffith.edu.au

Please complete the initial part of the survey and when directed open the video link of the video vignette and watch the video before completing the second part of the survey. The survey will take approximately 15 minutes to complete and the video will take between 15 and 25 minutes for a total time of approximately 40 minutes

Thank you once again

Tessa Fulton, Kerry Myatt, Garry Kirwan, Megan Dalton and Courtney Clark

1. Are you happy to proceed to the survey

- Yes
- No

* 2. Which video is this assessment related to?

- Apple
- Orange
- Grape
- Nectarine
- Raspberry
- Banana
- Strawberry
- Mango
- Melon
- Lime
- Avocado
- Blueberry

* 3. In which state do you currently practice?

- QLD
- NSW
- ACT
- VIC
- TAS
- SA
- WA
- NT

* 4. Which of the following best describes your primary clinical setting?

- Hospital (public)
- Hospital (private)
- Community based service
- Private practice
- University led clinic
- Non-government organisation (NGO)
- Other Government Department

* 5. What is the primary geographical setting for your clinical practice?

- ◯ Metropolitan
- ◯ Regional
- ◯ Rural
- ◯ Remote

* 6. How many years have you been clinically practicing as a physiotherapist?

- 0-2
- 3-5
- 6-8
- 9-11
- 12-15
- 15+

* 7. How many years experience do you have in paediatric clinical education?

- ◯ <1
- ◯ 1-3
- ◯ 4-6
- ◯ 7-9
- ◯ 10-12
- ◯ 12-14
- ◯ 14+

* 8. What is your level of confidence to assess student performance using the APP?

	Not Confident	Somewhat confident	Confident	Very confident
Select 1 of the following	◯	◯	◯	◯

Prior to watching the video we would like to set the following parameters to assist with assessing the student performance:

1. We understand that the APP is intended for assessment over time which is not possible in this context

2. Based on this when watching this video we ask you to base your global rating on the presumption that the student has been consistently performing an assessment and treatment similar to this level across the final week of placement

3. Therefore when answering the survey questions you are preparing for the summative assessment at the completion of the placement

Now click on the video link contained within the email you received and watch the video of the student performance in your designated clinical area. Make sure you keep this survey window open as you will be required to answer further questions upon completion of the video

* 9. Based on the video what is your global rating of the student performance?

Not Adequate	Adequate	Good	Excellent
○	○	○	○

10. For this performance to be marked as Adequate, please list 3-5 key performance indicators that you would have needed to see from the student:

11. For this performance to be marked as Good, please list 3-5 key performance indicators that you would have needed to see from the student:

12. What are 3-5 key performance indicators that you identified in this vignette to separate this performance from an Adequate marking?

* 13. Please rate the following statements

	Strongly Disagree	Disagree	Agree	Strongly Agree
The clinical scenario was realistic and believable	◯	◯	◯	◯
The video was professional and well presented	◯	◯	◯	◯

Unfortunately due to your selected years of experience you do not meet the inclusion criteria for this study. If you would like to discuss further with the research team please feel free to contact them on the details provided.

Thank you for your time and interest

Thank you for completing the blind assessing for this APP vignette!

We look forward to analysing the results and the videos will be available through the APPLinkup or by contacting the research team once the data has been collected.
